# DIETARY INTAKE AND SELF-REPORTED NUTRITION CONCERNS OF PEOPLE WITH PARKINSON’S AND THEIR INFORMAL CAREGIVERS

**DOI:** 10.1093/geroni/igz038.3404

**Published:** 2019-11-08

**Authors:** Dara L LoBuono, Kyla Shea, Sarah Sarah Dobiszewski, Alison Tovar, Skye N Leedahl, Furong Xu, Mahler Leslie, Ingrid E Lofgren

**Affiliations:** University of Rhode Island, Kingston, Rhode Island, United States

## Abstract

Dietary recommendations for managing Parkinson’s disease (PD) can be confusing for people with PD (PwPD) and their informal caregivers (ICGs) who often have the responsibility to buy and prepare foods. PwPD (63-78 years of age) and their ICGs (39-75 years of age) completed two 24-hour dietary recalls and semi-structured, dyadic interviews which were conducted to gather information about dyads’ (n=9) nutrition concerns. Calorie, macro- and micro-nutrient intake were averaged over two days and compared to the National Academy of Sciences’ dietary reference intakes. Independent t-tests and the Mann-Whitney U tests compared PwPD and ICG. Interviews were audio-recorded, transcribed verbatim, and analyzed for preliminary themes. Mean calorie intake was 1766.6±658.5 kcal/d; the majority of calories were from carbohydrates. Calorie and nutrient intake between PwPD and ICG did not differ. All participants were below recommendations for fiber (17.2±7.9g/d) and potassium (2213.3±890.0mg/d) and exceeded recommendations for sodium (2741.2±1396.9mg/d) and added-sugars (59.5 ±38.3g/d). More than half of participants (55.6%) agreed an eating plan to manage PD symptoms is important and that a nutrition consultation would be helpful. Emerging themes from qualitative interviews include: nutrition concerns related to PD symptoms, managing other conditions and PD, perceptions regarding diet quality, impact of diet on PD symptoms, and complementary medicine. In conclusion, there were no differences in dietary intake between PwPD and ICG, and participants presented with lack of adherence to recommended dietary reference intakes. Sub-optimal dietary intake and self-reported nutrition concerns strongly suggest including both nutrition professionals and ICGs when providing care for PwPD.

